# Role of Whole-Body Magnetic Resonance Imaging in Detecting Metastasis in Patients With Known Cancer

**DOI:** 10.7759/cureus.110651

**Published:** 2026-06-11

**Authors:** Navya Pentakota, Prabhu C S, Jeya Shambavi, Navin Kumar

**Affiliations:** 1 Department of Radiodiagnosis, Aarupadai Veedu Medical College and Hospital, Puducherry, IND; 2 Department of Pathology, Aarupadai Veedu Medical College and Hospital, Puducherry, IND

**Keywords:** cancer staging, computed tomography, diffusion-weighted imaging, metastasis, whole-body mri

## Abstract

Background: Cancer continues to impose a significant global health burden, with metastatic disease being the leading cause of cancer-related mortality. Accurate detection and staging of metastases are essential for prognostication and treatment planning. Conventional imaging modalities, including contrast-enhanced computed tomography (CECT), have limitations in detecting early or subtle metastatic lesions. Whole-body magnetic resonance imaging (WB-MRI), particularly with diffusion-weighted imaging (DWI), has emerged as a radiation-free technique that enables comprehensive evaluation with high soft-tissue contrast. Therefore, this study aimed to evaluate the diagnostic performance of WB-MRI, including DWI, in detecting nodal, skeletal, and visceral metastases in patients with known malignancies and to compare its performance with CECT on a patient-based analysis.

Methodology: This analytical cross-sectional study was conducted in the Department of Radiodiagnosis at Aarupadai Veedu Medical College and Hospital, Puducherry, over a period of six months. Eighty patients aged 18-60 years with histopathologically confirmed malignancy were included. All participants underwent CECT of the neck, thorax, and abdomen, followed by WB-MRI using a 1.5-T system with diffusion-weighted sequences. Imaging findings were documented and, wherever feasible, correlated with histopathology or cytology. Data were analyzed using descriptive statistics and the chi-square test in Statistical Package for the Social Sciences, version 26.0 (IBM Corp., Armonk, NY; 2019). A p value of <0.05 was considered statistically significant.

Results: The mean age of the participants was 53.39 ± 8.16 years, with a slight female predominance. Carcinoma of the cervix and breast carcinoma were the most common primary malignancies. WB-MRI demonstrated significantly higher sensitivity and specificity than CT in detecting nodal, skeletal, and visceral metastases. It showed excellent performance in detecting skeletal metastases and superior accuracy in identifying nodal and visceral involvement, with statistically significant differences compared to CT.

Conclusion: WB-MRI outperforms CT in the comprehensive detection of metastatic disease across multiple organ systems. Its high diagnostic accuracy, absence of ionizing radiation, and ability to detect early metastatic changes support its role as a reliable imaging modality for staging and follow-up in oncology.

## Introduction

Cancer remains a major global health burden, contributing substantially to morbidity and mortality worldwide. Despite advances in early detection and targeted therapies, metastatic disease continues to account for nearly 90% of cancer-related deaths [1]. Global estimates indicate a steady rise in both incidence and mortality, with approximately 19.3 million new cases and over 10 million deaths reported in 2020. In India, cancer incidence is projected to increase by nearly 12% over five years, with reported cases rising from approximately 1.16 million in 2018 to 1.39 million in 2020, along with a corresponding increase in mortality [2,3].

Metastasis involves the dissemination of malignant cells from a primary tumor to distant organs, often following characteristic patterns, such as skeletal involvement in breast and prostate cancers and visceral spread to the liver and lungs. Accurate detection and staging of metastatic disease are critical, as the extent and distribution of metastases strongly influence prognosis and therapeutic decision-making [4].

Imaging plays a central role in staging, treatment planning, and follow-up. Conventional approaches utilize multiple modalities, including radiography, ultrasonography, bone scintigraphy, computed tomography (CT), positron emission tomography/computed tomography (PET/CT), and regional magnetic resonance imaging (MRI), each with inherent limitations [5]. Whole-body magnetic resonance imaging (WB-MRI) has emerged as a comprehensive, radiation-free alternative that enables evaluation of the entire body in a single examination [6]. Its superior soft-tissue contrast and favorable safety profile make it particularly suitable for repeated assessments and for patients with renal impairment [7].

The addition of whole-body diffusion-weighted imaging (DWI), including DWI with background body signal suppression, has further enhanced lesion detection by providing functional information on tissue cellularity [8]. Comparative studies have demonstrated that WB-MRI has diagnostic performance comparable to PET/CT, with particular strength in detecting brain and liver metastases, while avoiding ionizing radiation and radioactive tracers [9].

Given the rising incidence of cancer and the critical need for accurate assessment of metastatic disease, there is an increasing demand for imaging techniques that combine whole-body coverage, high diagnostic accuracy, patient safety, and cost-effectiveness. WB-MRI, particularly when integrated with DWI, represents a promising solution to these challenges because it enables detection of early metastatic changes with excellent soft-tissue contrast while avoiding ionizing radiation. Therefore, the present study was designed to evaluate the diagnostic performance of WB-MRI for detecting nodal, skeletal, and visceral metastases in patients with histopathologically confirmed malignancies and to compare its sensitivity and specificity with those of contrast-enhanced computed tomography (CECT) using a patient-based diagnostic approach.

## Materials and methods

Study design and setting

This analytical, cross-sectional, hospital-based study was conducted in the Department of Radiodiagnosis at Aarupadai Veedu Medical College and Hospital, Puducherry. The study was carried out over a period of six months from July 2024 to December 2025 after obtaining approval from the Institutional Human Ethics Committee (IHEC No: AV/IHEC/01/2024/091). Written informed consent was obtained from all participants prior to enrollment.

Study population

A total of 80 patients aged between 18 and 60 years with histopathologically confirmed malignancy were included using purposive sampling. Patients willing to undergo both CECT and WB-MRI were enrolled in the study.

Patients who refused consent, pregnant women, individuals with known contrast allergy, and those with contraindications to MRI, such as metallic implants, cochlear implants, severe claustrophobia, or incompatible prosthetic devices, were excluded.

Sample size calculation

The sample size was calculated based on the study by Rashid et al. [10], assuming an expected WB-MRI sensitivity of 75%, relative precision of 16.7%, 5% level of significance, and 90% study power.

CT imaging protocol

All participants initially underwent CECT of the neck, thorax, and abdomen using a 16-slice GE Brivo CT scanner (GE Healthcare, Japan). Intravenous iodinated contrast material was administered in accordance with institutional imaging protocols. Axial images with multiplanar reconstructions were obtained during routine post-contrast acquisition phases appropriate for the suspected primary malignancy and metastatic evaluation.

WB-MRI protocol

WB-MRI was subsequently performed using a 1.5-T Philips Achieva DS MR system (Philips Healthcare, Brazil). Imaging was acquired from head to mid-thigh using coronal T1-weighted, T2-weighted, and diffusion-weighted imaging (DWI) sequences.

Diffusion-weighted imaging was performed using multiple b-values, and apparent diffusion coefficient (ADC) maps were generated for lesion characterization. Standard institutional oncologic WB-MRI acquisition parameters, including routine slice thickness and field-of-view settings, were maintained throughout the study. The average WB-MRI examination duration ranged between 35 and 45 minutes.

Image interpretation and reference standard

All imaging findings, including lesion location, size, morphology, and metastatic involvement, were recorded using a patient-based diagnostic assessment for nodal, skeletal, and visceral metastases. Experienced radiologists independently reviewed MRI and CT images.

Histopathological or cytological confirmation was used as the reference standard wherever feasible. In lesions where pathological confirmation could not be obtained, the final diagnosis was established using a composite reference standard based on clinical follow-up findings, conventional imaging correlation, and multidisciplinary oncologic assessment.

Statistical analysis

Statistical analysis was performed using IBM Statistical Package for the Social Sciences Statistics for Windows, version 26.0 (IBM Corp., Armonk, NY; 2019). Data normality was assessed using the Kolmogorov-Smirnov and Shapiro-Wilk tests. Descriptive statistics were expressed as frequencies, percentages, means, and standard deviations.

The diagnostic performance of WB-MRI and CT for detecting nodal, skeletal, and visceral metastases was evaluated using sensitivity and specificity. Comparative analysis between imaging modalities was performed, and a p value of <0.05 was considered statistically significant.

## Results

**Figure 1:**
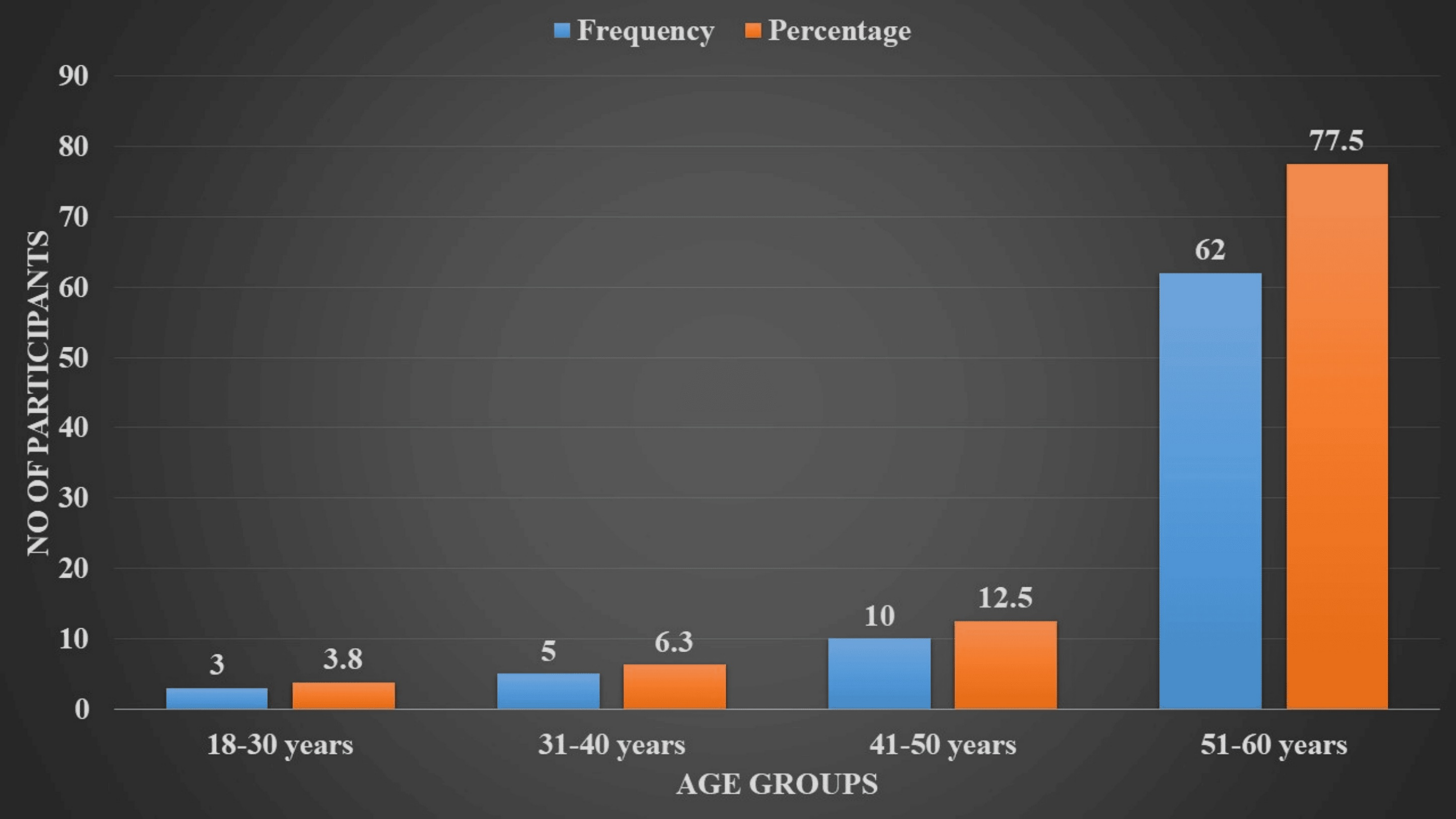
Age-wise distribution of the study participants

Overall, 62 (77.5%) participants were in the 51-60-year age group, indicating that the majority of the study population belonged to this age bracket, while younger age groups were less frequently represented. The mean age of the participants was 53.39 ± 8.16 years, with ages ranging from 22 to 60 years (Figure 1).

**Figure 2:**
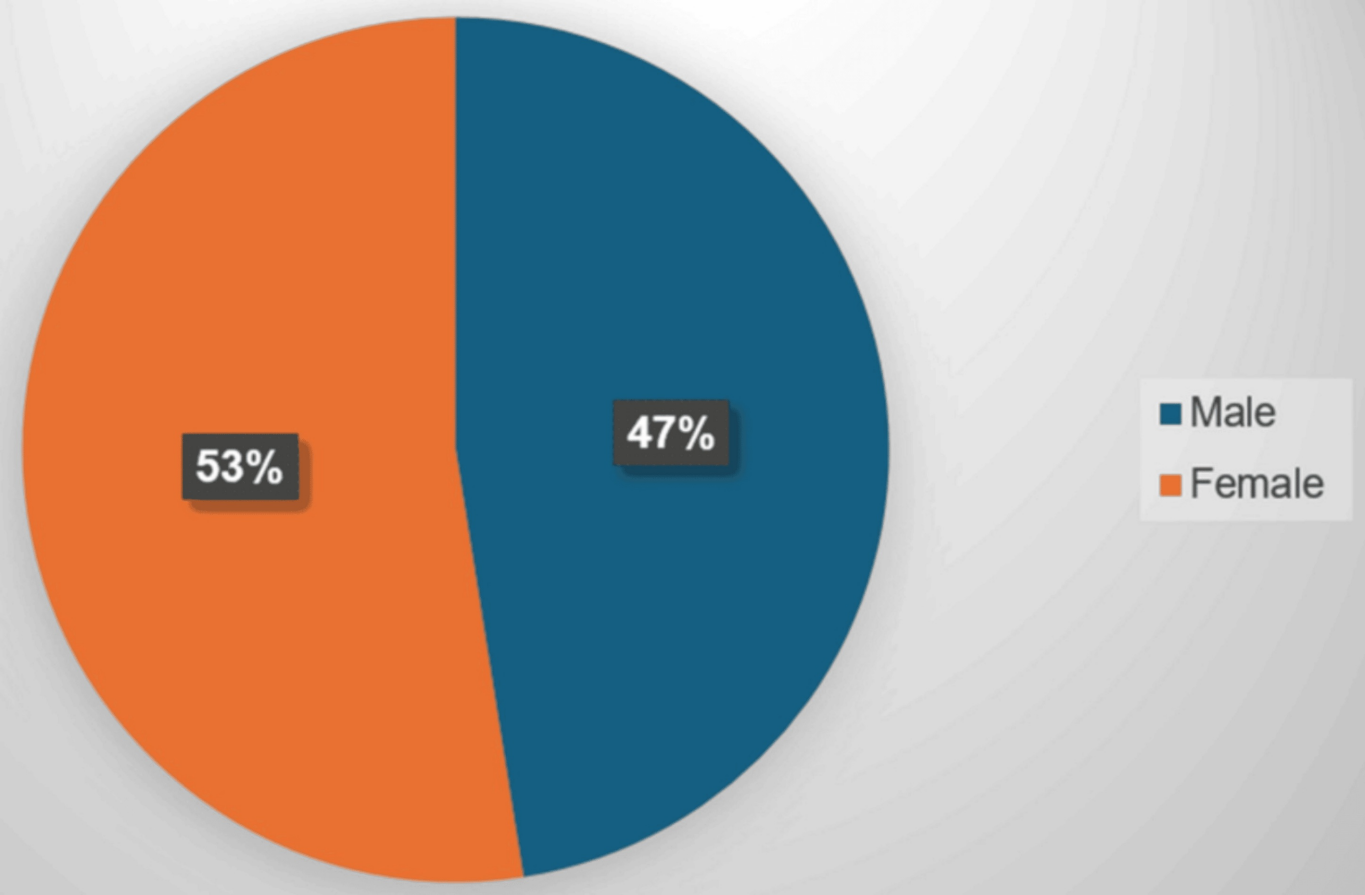
Gender distribution of the study participants

Regarding gender distribution, 42 (52.5%) were female participants, and 38 (47.5%) were male participants, indicating a slightly higher proportion of female participants in the study (Figure 2).

Cervical carcinoma was noted in 17 (21.3%) cases, followed by breast carcinoma in 13 (16.3%), hepatocellular and colorectal carcinoma each in nine (11.3%), lung carcinoma in seven (8.8%), renal cell carcinoma in six (7.5%), endometrial carcinoma in five (6.3%), prostate carcinoma in four (5.0%), pancreatic carcinoma and sarcoma each in three (3.8%), and thyroid and ovarian carcinoma each in two (2.5%) cases (Table 1).

**Table 1:** Types of primary lesion distribution among the study participants

Type of carcinoma	Total number, n (%)
Renal cell carcinoma	6 (7.5%)
Hepatocellular carcinoma	9 (11.3%)
Lung carcinoma	7 (8.8%)
Thyroid carcinoma	2 (2.5%)
Colorectal carcinoma	9 (11.3%)
Carcinoma cervix	17 (21.3%)
Breast carcinoma	13 (16.3%)
Pancreatic carcinoma	3 (3.8%)
Endometrial carcinoma	5 (6.3%)
Ovarian carcinoma	2 (2.5%)
Prostate carcinoma	4 (5%)
Sarcoma	3 (3.8%)

Carcinoma cervix was noted in 26 (22.4%) metastatic events, including nodal metastases in 11 (9.5%), skeletal metastases in three (2.6%), and visceral metastases in 12 (10.3%) cases. Breast carcinoma accounted for 19 (16.4%) metastatic events, while hepatocellular carcinoma was observed in 16 (13.8%) cases. Colorectal carcinoma accounted for 13 (11.2%) events, followed by lung carcinoma with 11 (9.5%) cases. Pancreatic carcinoma was seen in seven (6.0%) cases, and renal cell carcinoma in six (5.2%) cases. Endometrial carcinoma, prostate carcinoma, and sarcoma each accounted for five (4.3%) metastatic events. Endometrial carcinoma showed skeletal metastases in two (1.7%) cases and visceral metastases in three (2.6%) cases, with no nodal involvement. Prostate carcinoma demonstrated nodal metastases in three (2.6%) cases, skeletal metastases in one (0.9%), and visceral metastases in one (0.9%) case. Ovarian carcinoma had the least involvement, with only one (0.9%) nodal metastatic event and no skeletal or visceral metastases (Table 2).

**Table 2:** Distribution of metastasis by primary malignancies among the study participants ^*^Percentage of all metastatic events (n = 116) accounted for by that specific metastasis type from that primary ^†^Percentage of total metastatic events (n = 116) ^‡^% within that primary malignancy’s total metastases (i.e., row-wise percentage)

Primary malignancy	Nodal metastasis, n (%)^*^	Skeletal metastasis, n (%)^*^	Visceral metastasis, n (%)^*^	Total metastatic events, n (%)^†^
Carcinoma cervix	11 (9.5%) (42.3%^‡^)	3 (2.6%) (11.5%^‡^)	12 (10.3%) (46.2%^‡^)	26 (22.4%)
Breast carcinoma	8 (6.9%) (42.1%^‡^)	4 (3.4%) (21.1%^‡^)	7 (6.0%) (36.8%^‡^)	19 (16.4%)
Hepatocellular carcinoma	7 (6.0%) (43.8%^‡^)	4 (3.4%) (25.0%^‡^)	5 (4.3%) (31.3%^‡^)	16 (13.8%)
Colorectal carcinoma	6 (5.2%) (46.2%^‡^)	2 (1.7%) (15.4%^‡^)	5 (4.3%) (38.5%^‡^)	13 (11.2%)
Lung carcinoma	4 (3.4%) (36.4%^‡^)	2 (1.7%) (18.2%^‡^)	5 (4.3%) (45.5%^‡^)	11 (9.5%)
Pancreatic carcinoma	3 (2.6%) (42.9%^‡^)	2 (1.7%) (28.6%^‡^)	2 (1.7%) (28.6%^‡^)	7 (6.0%)
Renal cell carcinoma	5 (4.3%) (83.3%^‡^)	0 (0%)	1 (0.9%) (16.7%^‡^)	6 (5.2%)
Endometrial carcinoma	0 (0%)	2 (1.7%) (40%^‡^)	3 (2.6%) (60%^‡^)	5 (4.3%)
Prostate carcinoma	3 (2.6%) (60%^‡^)	1 (0.9%) (20%^‡^)	1 (0.9%) (20%^‡^)	5 (4.3%)
Sarcoma	2 (1.7%) (40%^‡^)	2 (1.7%) (40%^‡^)	1 (0.9%) (20%^‡^)	5 (4.3%)
Thyroid carcinoma	0 (0%)	1 (0.9%) (50%^‡^)	1 (0.9%) (50%^‡^)	2 (1.7%)
Ovarian carcinoma	1 (0.9%) (100%^‡^)	0 (0%)	0 (0%)	1 (0.9%)
Total	50 (100%)	23 (100%)	43 (100%)	116 (100%)

MRI demonstrated superior diagnostic performance compared with CT in detecting nodal metastasis. MRI showed a sensitivity of 80.6% and specificity of 75.9%, with true positives in 31 (53.4%) cases and true negatives in 19 (32.8%) cases, while false positives and false negatives were observed in five (8.6%) and three (5.2%) cases, respectively. In contrast, CT showed lower sensitivity (56.9%) and specificity (50.0%), with true positives in 29 (36.3%) cases and true negatives in seven (8.8%) cases, along with higher false positives and false negatives, each in 22 (27.5%) cases. The difference in diagnostic performance between the two modalities was statistically significant (Table 3).

**Table 3:** Association between CT and MRI of nodal metastasis ^*^Statistically significant at p value <0.05 CT: computed tomography; MRI: magnetic resonance imaging; df: degrees of freedom

Modality	True positive, n (%)	True negative, n (%)	False positive, n (%)	False negative, n (%)	Total, n	Sensitivity (%)	Specificity (%)	Chi-square (df)	p value
CT	29 (36.3)	7 (8.8)	22 (27.5)	22 (27.5)	80	56.9	50.0	8.21 (1)	0.004^*^
MRI	31 (53.4)	19 (32.8)	5 (8.6)	3 (5.2)	58	80.6	75.9	8.21 (1)	0.004^*^

MRI demonstrated superior diagnostic performance compared with CT. MRI showed a sensitivity of 100.0% and specificity of 100.0%, with 14 (100%) true positives and 52 (100%) true negatives, with no false positives or false negatives. In contrast, CT demonstrated lower sensitivity (50.0%) and specificity (78.8%), with true positives in 14 (50.0%) cases and true negatives in 52 (78.8%) cases, along with false positives in 14 (50.0%) cases and false negatives in 14 (21.2%) cases. The difference in diagnostic performance between the two modalities was statistically significant (p = 0.001) (Table 4).

**Table 4:** Diagnostic performance of CT and MRI in detecting skeletal metastasis ^*^Percentages for TP and FP are column-wise within total predicted positives for each modality ^†^Percentages for TN and FN are column-wise within total predicted negatives for each modality ^**^Statistically significant at p value <0.05 CT: computed tomography; MRI: magnetic resonance imaging; TP: true positive; FP: false positive

Variable	CT	MRI
True positive, n (%)^*^	14 (50.0%)	14 (100%)
False positive, n (%)^*^	14 (50.0%)	0 (0%)
Total predicted positive	28	14
True negative, n (%)^†^	52 (78.8%)	52 (100%)
False negative, n (%)^†^	14 (21.2%)	0 (0%)
Total predicted negative	66	52
Sensitivity (%)	50.0%	100.0%
Specificity (%)	78.8%	100.0%
p value	0.001^**^	0.001^**^

MRI demonstrated better diagnostic performance than CT in detecting visceral metastasis. MRI showed a sensitivity of 82.9% and specificity of 84.2%, with true positives in 41 (97.6%) cases and true negatives in two (33.3%) cases, while false positives and false negatives were observed in one (2.4%) and four (66.7%) cases, respectively. In comparison, CT demonstrated lower sensitivity (69.0%) and specificity (71.1%), with true positives in 31 (88.6%) cases and true negatives in 12 (92.3%) cases, along with false positives in four (11.4%) cases and false negatives in one (7.7%) case. The difference in diagnostic performance between the two modalities was statistically significant (Table 5).

**Table 5:** Association between CT and MRI of visceral metastasis ^*^Statistically significant at p value <0.05 CT: computed tomography; MRI: magnetic resonance imaging

Modality	True positive, n (%)	False positive, n (%)	Total predicted positive	True negative, n (%)	False negative, n (%)	Total predicted negative	Sensitivity (%)	Specificity (%)	Fisher p value
CT	31 (88.6)	4 (11.4)	35	12 (92.3)	1 (7.7)	13	69.0	71.1	0.001^*^
MRI	41 (97.6)	1 (2.4)	42	2 (33.3)	4 (66.7)	6	82.9	84.2	0.001^*^

## Discussion

In the present study, the age distribution showed a clear predominance of older adults, with the highest proportion in the 51-60 years age group (77.5%), while younger age groups were minimally represented. Participants aged 41-50 years accounted for 12.5%, whereas those aged 40 years or less constituted less than 11% of cases. The mean age of the participants was 53.39 ± 8.16 years. Rashid et al. [10] reported similar findings, noting that the majority of patients undergoing WB-MRI for metastatic workup were above 50 years of age, reflecting the increasing cancer burden with advancing age. In contrast, Sung et al. [11] reported a higher proportion of patients below 50 years undergoing WB-MRI for colorectal cancer, which differs from the present study.

In the present study, a slight female predominance was observed, with female patients accounting for 52.5% of the study population compared with 47.5% male patients. Hoveling et al. [12] reported similar findings, indicating a higher prevalence of malignancies among female patients. In contrast, Rashid et al. [10] reported a higher prevalence of malignancies among male patients.

The present study demonstrated a heterogeneous distribution of primary malignancies, with carcinoma of the cervix constituting the most common primary tumor (21.3%), followed by breast carcinoma (16.3%). Hepatocellular carcinoma and colorectal carcinoma were equally represented (11.3% each), while lung carcinoma accounted for 8.8% of cases. Renal cell carcinoma, endometrial carcinoma, and prostate carcinoma contributed a moderate proportion, whereas pancreatic carcinoma, sarcoma, thyroid carcinoma, and ovarian carcinoma were less frequently encountered. Rashid et al. [10] similarly reported breast malignancy as the most frequent primary tumor among patients undergoing WB-MRI for metastatic assessment. In contrast, Planchard et al. [13] identified lung carcinoma as a leading indication for WB-MRI due to its aggressive behavior and high propensity for early metastatic spread. The relatively lower proportion of lung carcinoma in the present study may reflect regional differences in cancer prevalence, screening practices, referral patterns, and the higher burden of gynecological malignancies in the study population. WB-MRI is widely used for initial staging and follow-up assessment across a broad spectrum of malignancies, particularly those with a high propensity for spread to the skeleton, central nervous system, and abdominal viscera, including breast and colorectal cancers [14].

In the present study, MRI demonstrated superior diagnostic performance compared to CT in detecting nodal metastases. CT showed relatively low sensitivity (56.9%) and specificity (50.0%), with a high number of both false-positive and false-negative results, indicating limited reliability in accurately identifying nodal involvement. In contrast, MRI achieved higher sensitivity (80.6%) and specificity (75.9%), reflecting its improved ability to detect metastatic lymph nodes while reducing misclassification. The difference in diagnostic accuracy between CT and MRI was statistically significant (p = 0.004), underscoring MRI’s advantage in nodal assessment. Lecouvet et al. [15] reported similar findings, demonstrating that WB-MRI, particularly with diffusion-weighted imaging, has higher sensitivity than CT for detecting nodal metastases, as it assesses tissue characteristics rather than relying solely on nodal size. In contrast, Kwee and Kwee [16] reported that although MRI can detect subcentimetric metastatic lymph nodes missed on CT, it may have lower specificity, as reactive or inflammatory nodes can mimic metastatic disease.

In the present study, MRI demonstrated markedly superior diagnostic performance compared to CT in detecting skeletal metastases. All CT-positive cases were also identified on MRI, and MRI additionally detected several metastatic lesions missed on CT, resulting in a sensitivity of 100% for MRI compared with 50.0% for CT. MRI specificity was also higher (100%) than CT (78.8%), indicating an excellent ability to exclude skeletal metastasis when absent correctly. The difference in diagnostic performance between CT and MRI was statistically significant (p = 0.001), further supporting MRI’s advantage in skeletal evaluation. Rashid et al. [10] reported similar findings, showing that WB-MRI has higher sensitivity and diagnostic accuracy than CT for detecting skeletal metastases, particularly early marrow and small focal lesions that are often missed on CT. Similarly, studies in lung cancer patients have confirmed MRI’s superior lesion-by-lesion detection due to its excellent soft-tissue and marrow contrast. In contrast, Koh and Collins [17] demonstrated that quantitative ADC values from diffusion-weighted MRI can help differentiate malignant from benign marrow changes, thereby reducing false-positive findings that may occur with qualitative assessment alone.

In the present study, MRI also demonstrated superior diagnostic performance compared to CT in detecting visceral metastases. CT showed lower sensitivity (69.0%) and specificity (71.1%), with a higher number of false-positive and false-negative results, indicating limited accuracy in identifying visceral metastatic lesions. In contrast, MRI achieved higher sensitivity (82.9%) and specificity (84.2%), reflecting improved detection and reduced misclassification. The difference in diagnostic performance between CT and MRI was statistically significant (p = 0.001), highlighting MRI’s advantage in visceral assessment. Schmidt et al. [14] reported similar findings, demonstrating that WB-MRI has higher sensitivity and diagnostic accuracy than CT for detecting visceral metastases, particularly small or early lesions that may be missed on CT due to limited soft-tissue contrast. In contrast, Antoch et al. [18] reported that quantitative ADC measurements from diffusion-weighted MRI can improve differentiation between malignant and benign visceral lesions, thereby reducing false-positive findings and enhancing diagnostic confidence.

While CT remains useful for assessing gross anatomical changes, it has limitations in detecting small, early, or nonenlarged metastatic lesions. Overall, these findings highlight the clinical value of WB-MRI as a reliable modality for comprehensive metastatic evaluation, supporting its use in precise staging, treatment planning, and follow-up in oncology patients.

Limitations

This study has several limitations that should be considered while interpreting the findings. First, the study was conducted at a single center with a relatively modest sample size, which may limit the generalizability of the results. The use of purposive sampling may have introduced selection bias, and the study population was predominantly within the 51-60-year age group, with exclusion of patients older than 60 years. Second, the inclusion of multiple primary malignancies introduced heterogeneity in metastatic patterns and disease behavior, which may influence diagnostic comparisons across imaging modalities.

Although histopathological or cytological confirmation was used wherever feasible, not all metastatic lesions could undergo tissue confirmation due to practical and ethical considerations. Therefore, a composite reference standard based on clinical and imaging follow-up was used in selected cases, which may have introduced verification bias. Additionally, interobserver variability and reproducibility analysis were not assessed, and image interpretation was performed within a routine clinical setting. The study also did not evaluate workflow-related factors such as cost-effectiveness, scan duration, patient tolerability, or accessibility of WB-MRI in resource-limited settings.

Furthermore, while WB-MRI demonstrated excellent diagnostic performance, particularly for skeletal metastases, these findings should be interpreted cautiously, considering the relatively small sample size and patient-based analytical approach. Larger multicenter studies with standardized imaging protocols, blinded interpretation, and uniform pathological confirmation are recommended to validate these findings further.

## Conclusions

The present study demonstrated that WB-MRI showed higher diagnostic sensitivity and specificity than CT for detecting nodal, skeletal, and visceral metastases in patients with known primary malignancies. The superior soft-tissue contrast and diffusion-weighted imaging capability of WB-MRI contributed to improved detection of metastatic lesions, particularly skeletal and soft-tissue involvement. These findings suggest that WB-MRI may serve as a promising radiation-free imaging modality for comprehensive metastatic assessment and follow-up in oncology patients.

However, the findings should be interpreted cautiously, considering the relatively modest sample size, single-center design, incomplete histopathological confirmation in all lesions, and methodological limitations inherent to the study. Larger multicenter studies with standardized imaging protocols and uniform reference standards are required to further validate the diagnostic performance and clinical applicability of WB-MRI across different oncologic settings.

## References

[1] Guan X (2015). Cancer metastases: challenges and opportunities. Acta Pharm Sin B.

[2] Shafi L, Iqbal P, Khaliq R (2023). Cancer burden in India: a statistical analysis on incidence rates. Indian J Public Health.

[3] Mathur P, Sathishkumar K, Chaturvedi M (2020). Cancer statistics, 2020: report from National Cancer Registry Programme, India. JCO Glob Oncol.

[4] Guimarães MD, Noschang J, Teixeira SR (2017). Whole-body MRI in pediatric patients with cancer. Cancer Imaging.

[5] Godinho MV, Lopes FP, Costa FM (2018). Whole-body magnetic resonance imaging for the assessment of metastatic breast cancer. Cancer Manag Res.

[6] Schmidt GP, Haug AR, Schoenberg SO, Reiser MF (2006). Whole-body MRI and PET-CT in the management of cancer patients. Eur Radiol.

[7] Bisschop C, de Heer EC, Brouwers AH, Hospers GA, Jalving M (2020). Rational use of (18)F-FDG PET/CT in patients with advanced cutaneous melanoma: a systematic review. Crit Rev Oncol Hematol.

[8] Li B, Li Q, Nie W, Liu S (2014). Diagnostic value of whole-body diffusion-weighted magnetic resonance imaging for detection of primary and metastatic malignancies: a meta-analysis. Eur J Radiol.

[9] Kwee TC, Takahara T, Ochiai R, Nievelstein RA, Luijten PR (2008). Diffusion-weighted whole-body imaging with background body signal suppression (DWIBS): features and potential applications in oncology. Eur Radiol.

[10] Rashid RJ, Tahir SH, Kakamad FH (2023). Whole‑body MRI for metastatic workup in patients diagnosed with cancer. Mol Clin Oncol.

[11] Sung H, Siegel RL, Laversanne M (2025). Colorectal cancer incidence trends in younger versus older adults: an analysis of population-based cancer registry data. Lancet Oncol.

[12] Hoveling LA, Eijkelboom A, Schuurman M (2025). Disparities in cancer incidence and stage at diagnosis between male and female patients in the Netherlands. Eur J Cancer.

[13] Planchard D, Popat S, Kerr K (2018). Metastatic non-small cell lung cancer: ESMO clinical practice guidelines for diagnosis, treatment and follow-up. Ann Oncol.

[14] Schmidt GP, Reiser MF, Baur-Melnyk A (2009). Whole-body MRI for the staging and follow-up of patients with metastasis. Eur J Radiol.

[15] Lecouvet FE, Talbot JN, Messiou C, Bourguet P, Liu Y, de Souza NM (2014). Monitoring the response of bone metastases to treatment with magnetic resonance imaging and nuclear medicine techniques: a review and position statement by the European Organisation for Research and Treatment of Cancer imaging group. Eur J Cancer.

[16] Kwee RM, Kwee TC (2019). Whole-body MRI for preventive health screening: a systematic review of the literature. J Magn Reson Imaging.

[17] Koh DM, Collins DJ (2007). Diffusion-weighted MRI in the body: applications and challenges in oncology. AJR Am J Roentgenol.

[18] Antoch G, Vogt FM, Freudenberg LS (2003). Whole-body dual-modality PET/CT and whole-body MRI for tumor staging in oncology. JAMA.

